# Genome-Wide Comparative Functional Analyses Reveal Adaptations of *Salmonella* sv. Newport to a Plant Colonization Lifestyle

**DOI:** 10.3389/fmicb.2018.00877

**Published:** 2018-05-18

**Authors:** Marcos H. de Moraes, Emanuel Becerra Soto, Isai Salas González, Prerak Desai, Weiping Chu, Steffen Porwollik, Michael McClelland, Max Teplitski

**Affiliations:** ^1^Soil and Water Sciences Department, University of Florida, Gainesville, FL, United States; ^2^Center for Genomic Sciences, National Autonomous University of Mexico, Cuernavaca, Mexico; ^3^Department of Biology, The University of North Carolina at Chapel Hill, Chapel Hill, NC, United States; ^4^Howard Hughes Medical Institute, University of North Carolina at Chapel Hill, Chapel Hill, NC, United States; ^5^Curriculum in Bioinformatics and Computational Biology, University of North Carolina at Chapel Hill, Chapel Hill, NC, United States; ^6^Department of Microbiology and Molecular Genetics, University of California, Irvine, Irvine, CA, United States

**Keywords:** tomato, plant-microbe interactions, comparative genomics, pan-genome, vegetable safety

## Abstract

Outbreaks of salmonellosis linked to the consumption of vegetables have been disproportionately associated with strains of serovar Newport. We tested the hypothesis that strains of sv. Newport have evolved unique adaptations to persistence in plants that are not shared by strains of other *Salmonella* serovars. We used a genome-wide mutant screen to compare growth in tomato fruit of a sv. Newport strain from an outbreak traced to tomatoes, and a sv. Typhimurium strain from animals. Most genes in the sv. Newport strain that were selected during persistence in tomatoes were shared with, and similarly selected in, the sv. Typhimurium strain. Many of their functions are linked to central metabolism, including amino acid biosynthetic pathways, iron acquisition, and maintenance of cell structure. One exception was a greater need for the core genes involved in purine metabolism in sv. Typhimurium than in sv. Newport. We discovered a gene, *papA*, that was unique to sv. Newport and contributed to the strain’s fitness in tomatoes. The *papA* gene was present in about 25% of sv. Newport Group III genomes and generally absent from other *Salmonella* genomes. Homologs of *papA* were detected in the genomes of *Pantoea, Dickeya*, and *Pectobacterium*, members of the Enterobacteriacea family that can colonize both plants and animals.

## Introduction

Salmonellosis outbreaks linked to the consumption of fruits (tomatoes, cucumbers, cantaloupes), leafy green vegetables and sprouts became an important public health issue over the last decade, defying the traditional notion that this pathogen is only associated with products of animal origin (Teplitski et al., 2009; Hernandez-Reyes and Schikora, 2013; Jackson et al., 2013; Wiedemann et al., 2014; Bennett et al., 2015). A CDC study identified *Salmonella* sv. Newport as the predominant serovar involved in outbreaks traced to vegetables. It was responsible for 57% of outbreaks associated with all fresh vegetables, and for 29% of outbreaks associated with vine-stalk vegetables (such as tomatoes and cucumbers). Tomatoes were a major source of salmonellosis outbreaks, and were implicated in up to 90% of outbreaks linked to vine-stalk vegetables, with sv. Newport responsible for 32% of them (Jackson et al., 2013). An analysis of outbreak occurrence from 1990 to 2010 identified 15 outbreaks that were associated with fresh tomatoes, and sv. Newport was responsible for six of them (Bennett et al., 2015). The association of salmonellosis outbreaks with sv. Newport and fresh vegetables reported in independent geographical locations suggests that this serovar may have a close relationship with plants and/or has evolved to persist in the vegetable production environment. At least three hypotheses can explain this phenomenon.

One hypothesis for the overrepresentation of sv. Newport in produce-associated outbreaks is that it evolved functions that make it more fit in plants. This hypothesis is supported by the evidence that sv. Newport outcompetes other serovars during plant colonization. When tested for proliferation in tomatoes, *Salmonella* sv. Newport reached higher cell numbers in green and pink tomatoes than sv. Typhimurium, Braenderup, and Montevideo (Marvasi et al., 2013b). Cells of serovar Newport were also recovered at higher rates from tomato rhizosphere than those of the serovars Saintpaul, Typhimurium, and Montevideo (Zheng et al., 2013). At least in part, the ability of some strains of sv. Newport to be more competitive within tomatoes could be due to spontaneous non-*rdar* mutations, which increased *Salmonella* fitness inside tomatoes (Zaragoza et al., 2012).

Secondly, strains of serovar Newport may be more fit in plants than those of other serovars because they are better able to adapt their physiology to enter and proliferate in tomato tissues (Teplitski and de Moraes, 2018). Studies with regulatory mutants and mutants in metabolic pathways suggest that during tomato colonization, *Salmonella* utilizes carbohydrates and inorganic sources of nitrogen and then uses the acquired nutrients to synthesize amino acids, LPS, and capsule. For example, genes involved in amino acid biosynthesis and iron acquisition were important for *Salmonella* growth in tomato pericarps. Inside tomato fruits, at least 51 *Salmonella* genes were differently regulated, including *fadH*, involved in fatty acid degradation, and *cysB*, the regulator for cysteine biosynthesis and acquisition. Changes in surface structures are also part of the *Salmonella* strategy for proliferation within plant tissues. In unripe tomatoes, the *yihT* gene, involved in the synthesis of O-antigen, is required for successful colonization of pericarps (Noel et al., 2010; Marvasi et al., 2013a; Nugent et al., 2015; de Moraes et al., 2017). These observations suggest that the environment in plant hosts is nutritionally unbalanced, and *Salmonella* has to employ and coordinate a diverse set of functions to thrive and establish itself within a niche already occupied by the native microbiota. In many respects, these observations are in line with the requirements of other phytobacteria during their interactions with plants (Lindow and Leveau, 2002; Lindow and Brandl, 2003). However, an earlier analysis of the totality of functions used by *S.* Typhimurium to colonize tomatoes revealed that, despite some similarities with phytobacteria in its plant colonization strategies, it relies on a distinct set of functions to establish itself within tomatoes (de Moraes et al., 2017).

Lastly, the advantage of sv. Newport over other serovars in colonization of plants could be enhanced by the presence of intrinsic colonization factors in this *Salmonella* clade. Studies of *Salmonella* virulence in animals show how the serovars’ genetic diversity can affect interactions with the hosts. For example, distribution of virulence plasmids and pathogenicity islands is serovar-specific, and the presence of these genetic determinants appears to impact host range (Barrow et al., 1987; Barrow and Lovell, 1988; Libby et al., 1997; Desai et al., 2013). Is it possible that sv. Newport has functions, not commonly present in other serovars of *Salmonella*, that allow it to colonize plants more efficiently?

In this study, we applied comparative genomics combined with transposon insertion screening analysis to identify unique features that distinguish sv. Newport from sv. Typhimurium in the *Salmonella*-tomato interaction model.

## Materials and Methods

### Bacterial Strains and Culture Conditions

Bacterial strains and plasmids used in this study are listed in **Table 1**. *Salmonella enterica* and *Escherichia coli* strains were propagated in LB (Luria Bertani) broth (Fisher Scientific) at 37 or 30°C, as specified in the text. When necessary, bacteria were plated onto LB agar (Fisher Scientific) or XLD (Oxoid) agar plates. As appropriate, growth media were supplemented with 100 μg/ml ampicillin, 60 μg/ml kanamycin or 20 μg/ml chloramphenicol. Construction of mutants was done using λ Red mutagenesis (Datsenko and Wanner, 2000). Typically, entire ORFs (from the start to the stop codon) were excised and replaced with a frt-*kan*-frt or a frt-*cat*-frt cassette, and the kanamycin (or chloramphenicol) resistance gene was flipped out as in Datsenko and Wanner (2000).

**Table 1:** Strains used in this study.

Strain	Genotype	Source or
name		construction
C4.2	*Salmonella* sv. Newport C4.2	*Salmonella* sv. Newport isolate from a tomato field in Virginia owned by DiMare
ISG9	*Salmonella* sv. Newport C4.2 ppeg.4639::FRT-*cm*-FRT	Constructed using Datsenko and Wanner mutagenesis
ISG7	*Salmonella* sv. Newport C4.2 *phoN*-FRT-*cm*-FRT	Strain carrying a FRT-*cm*-FRT insertion downstream of *phoN*
ISG10	*Salmonella* sv. Newport C4.2 *glnA*::FRT-*cm*-FRT	Constructed using Datsenko and Wanner mutagenesis
ISG11	*Salmonella* sv. Newport C4.2 peg.4132::FRT-*cm*-FRT	Constructed using Datsenko and Wanner mutagenesis
ISG12	*Salmonella* sv. Newport C4.2 peg.4640::FRT-*cm*-FRT	Constructed using Datsenko and Wanner mutagenesis
ISG13	*Salmonella* sv. Newport C4.2 *ilvD*::FRT-*cm*-FRT	Constructed using Datsenko and Wanner mutagenesis
ISG14	*Salmonella* sv. Newport C4.2 *metA*::FRT-*cm*-FRT	Constructed using Datsenko and Wanner mutagenesis
ISG15	*Salmonella* sv. Newport C4.2 peg.4638::FRT-cm-FRT	Constructed using Datsenko and Wanner mutagenesis
ISG16	*Salmonella* sv. Newport C4.2 *glnG*::FRT-*cm*-FRT	Constructed using Datsenko and Wanner mutagenesis
ISG17	*Salmonella* sv. Newport C4.2 *thrC*::FRT-cm-FRT	Constructed using Datsenko and Wanner mutagenesis
ISG19	*Salmonella* sv. Newport C4.2 *ΔpapA phoN::papA*	Constructed using Datsenko and Wanner mutagenesis

### Transposon Insertion Library Screening in Tomato Pericarps

Tomato inoculation with a *S. enterica* Newport C4.2 transposon library was performed as previously done with *S. enterica* Typhimurium ATCC14028 (de Moraes et al., 2017). Tomatoes (cultivar Campari) were obtained from a local grocery store, where they are sold on the vine in a clam shell plastic container. The *S. enterica* sv. Newport transposon library was grown overnight in LB with the addition of kanamycin at 37°C, and the resulting cultures were resuspended in PBS. Approximately 10^8^ CFU/ml in 3 μL of PBS were inoculated into three shallow wounds in tomato pericarps, and six fruits were used. Tomato fruits were incubated at 22°C and a relative humidity of ∼60% for 7 days. Under these conditions, no gross changes in the appearance of the fruit were observed, although ripening clearly progressed. *Salmonella* was recovered by collecting 1 g samples of tomato tissue around ∼1 cm of the inoculation site; samples from the same fruit were combined and homogenized in a stomacher (Sevard). *Salmonella* cells were recovered by centrifugation and were then resuspended and cultured in 50 ml of LB broth for 6 h at 37°C and 250 rpm.

### Transposon Insertion Library Construction and Analysis

The transposon insertion library construction and analysis in sv. Newport were performed as described before (de Moraes et al., 2017). Briefly, a library of *S. enterica* serovar Newport C4.2 Tn5 insertion mutants was constructed with a mini-Tn5 derivative into which we inserted an N_18_ random barcode using PCR. This derivative was integrated into the genome using the EZ-Tn5 < T7/KAN-2 > promoter insertion kit (Epicentre Biotechnologies, Madison, WI, United States). The transposome complex was dialyzed against water before electroporation into fresh electrocompetent cells of *S. enterica* sv. Newport C4.2. Transformed cells were recovered on LB agar with kanamycin after overnight growth at 37°C.

Mapping of barcoded transposons to specific locations in the genome was performed as described before (de Moraes et al., 2017). Briefly, genomic DNA from the library was extracted using the GenElute bacterial genomic DNA kit (Sigma-Aldrich). DNA was fragmented by sonication and ligated to Illumina primers. This product was used to amplify the regions, including the N_18_ barcode and the genomic DNA adjacent to the transposon insertion, using a stepwise nested PCR (de Moraes et al., 2017). Resulting amplicons were purified with the QIAquick PCR Product Purification kit (Qiagen), and 150-base reads were obtained at both ends. The resulting reads were mapped against the *de novo* assembled sv. Newport C4.2 genome (see below) using Bowtie2. The N_18_ barcode tag for each mapped read was identified using custom Perl scripts. The same reads, trimmed to remove Tn5 sequences, were also employed to assemble the sv. Newport C4.2 genome, which was then annotated using the RAST package^1^.

For experiments in tomatoes, transposons were quantified as before (de Moraes et al., 2017). In brief, bacteria were recovered from tomatoes and grown in LB+60 μg/ml kanamycin. Bacteria were pelleted, lysed and subjected to PCR using primers directly flanking the N_18_ barcode. The frequency of each barcode was enumerated by Illumina sequencing of 20 bases. The aggregated abundances for the input and output libraries were statistically analyzed using edgeR, and the log_2_-fold changes and FDRs were reported.

### Genome Sequence Retrieval, Quality Control and Assembly

Raw reads from Illumina sequencing of *S. enterica* sv. Newport and sv. Typhimurium strains were recovered from the NCBI Sequence Read Archive (SRA). The SRA identifiers of the strains used are listed in **Supplementary Table S1**. We opted to assemble genomes *de novo* to remove biases associated with different assembly methods and to employ the same quality standards. The genomes of the type strains (GCA_000022165.1, GCA_000016045.1, and GCA_000171415.1) were recovered from the NCBI Genome databases. Read quality control and visualization were done using the package Trim Galore (Andrews et al., 2015). Genome assembly was done using SPAdes, using default parameters (Bankevich et al., 2012). Assessment of the genome assembly quality was done with CheckM (Parks et al., 2015). Genomes with more than 1% of contamination and less than 99% of completeness were excluded. Prokka was used to annotate genomes (Seemann, 2014). The resulting.gff files were fed into Roary to build the pan-genome matrix (Page et al., 2015). The analysis of the pan-genome matrix was performed using *ad hoc* R scripts. We used power-law regression to model the total size of the *Salmonella* pan-genome. To that end, analysis of random permutations of the addition of new genomes was performed and the number of new genes found per addition was recorded, and used in the regression to estimate the expansion of the pan-genome.

### Competition Assays

To confirm the results from the transposon insertion sequencing analysis, the fitness of individual isogenic mutants in relation to the wild-type was estimated. The bacterial population densities of overnight cultures of the wild type strain sv. Newport C4.2 and an isogenic mutant built in this background were set to similar numbers by adjusting their OD_600_ to the same level. Cells were then spun and resuspended in PBS to the original volume and mixed in a 1:1 ratio, followed by a 10,000-fold dilution in PBS. 3 μl of this mix were inoculated into the tomato pericarp in three separate wounds resulting in ∼10^3^ CFU per tomato fruit. To get an accurate count of the wild type and mutant cells in the inoculum, an aliquot was serially diluted and plated onto XLD agar and incubated overnight at 37°C; fifty randomly picked colonies were then patched onto LB agar with chloramphenicol and the initial mutant:wild-type ratio was determined.

The inoculated tomatoes were incubated at 22°C for 7 days. Wound sites were then sampled using a sterile loop and *Salmonella* cells were recovered from a streak on XLD plates. Fifty colonies were patched onto LB agar with chloramphenicol to determine the mutant:wild-type ratio in the recovered sample. The competition index (CI) was calculated using the formula (MUT_out_:WT_out_)/(MUT_in_:WT_in_) (Noel et al., 2010). Statistical significance was determined using ANOVA against the CI of the neutral mutant ISG7. This neutral mutant was constructed with a FRT-*cm*-FRT insertion downstream of *phoN* known to not affect *Salmonella* fitness in tomatoes (Cox et al., 2013). The neutral phenotype of ISG7 was confirmed by competitions against the wild type. The software JMP version 12 was used for all CI analyses.

### Growth Curve and *rdar* Phenotype Characterization

Responses of the strains to oxidative stress were compared by diluting overnight cultures to OD_600_ = 0.01 in 3 ml of LB with or without 0.5 mM paraquat. The cultures were incubated at 37°C with shaking at 200 rpm. 100 μl aliquots were collected hourly to estimate culture concentrations by serial dilution and plating onto LB agar. All experiments were replicated three times. The *rdar* phenotype was evaluated by spotting 5 μl of an overnight culture onto salt-less LB with Congo Red as described by Zaragoza et al. (2012).

## Results and Discussion

### Clade Separation Using Phylogenetic Analysis

**FIGURE 1:**
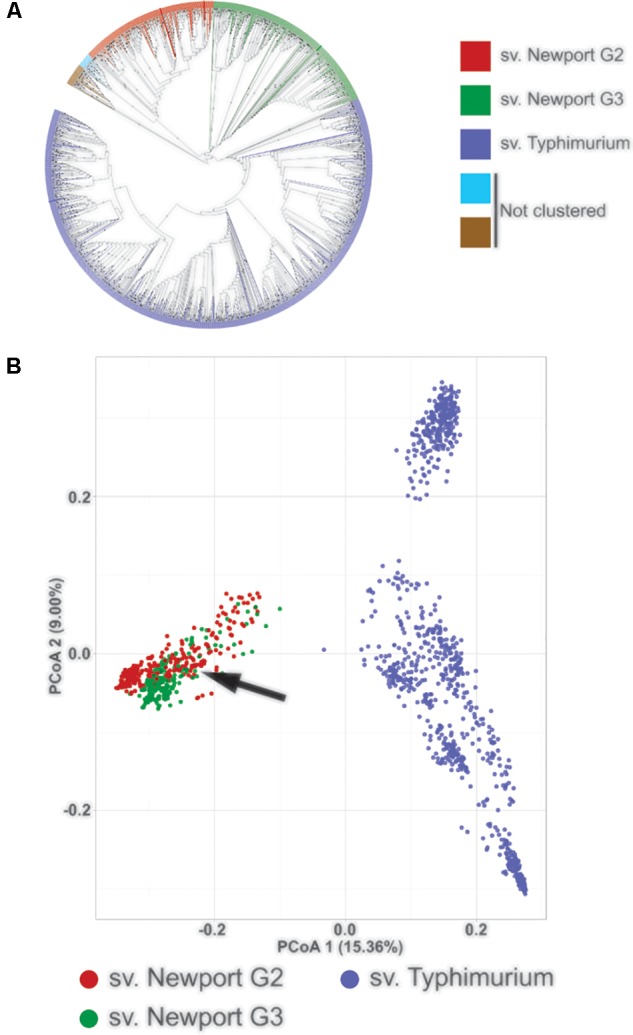
Phylogenetic and comparative genomic analysis of the sv. Typhimurium and sv. Newport genomes. **(A)** Phylogenetic tree for 1,597 genomes of sv Typhimurium and sv Newport isolates, constructed using SNPs and minimal evolution over raw distance. Colors represent the major clades identified and dots represent branches with bootstrap values higher than 0.85. iTOL v4 (Letunic and Bork, 2016) was used to visualize the tree. **(B)** Principal Coordinate Analysis of the gene presence and absence profile of the same genomes. *Salmonella* sv. Typhimurium strains are represented in purple, sv. Newport Group II isolates in red and sv. Newport Group III strains in green. The arrow indicates strain C4.2 of sv. Newport, recovered from a tomato-linked outbreak of human salmonellosis and used in this study.

In this study, we compared the genes required for growth of an *S. enterica* sv. Newport strain in tomatoes to previous results (de Moraes et al., 2017) obtained for a Typhimurium strain. We first used comparative genomics analysis to determine whether the strain of sv. Newport recovered from a tomato-linked outbreak and used in this study is an outlier within serovar Newport or whether it is a typical representative. A phylogenetic analysis, constructed with minimum-evolution using SNP distances obtained from 1526 *Salmonella* genomes (**Supplementary Table S1**), formed distinct clades for sv. Typhimurium and sv. Newport and displayed a small number of fast evolving genomes with no clear grouping (**Figure 1A** and **Supplementary Figure S1**). This result is consistent with previous *Salmonella* phylogenetic analyses, supporting the notion that sv. Typhimurium strains group as one clade and serovar Newport strains are divided into three different clades (Group I, Group II, Group III) (Sangal et al., 2010).

We identified the sv. Newport clades obtained in our analysis by placing the sv. Newport type strains SL257, known to be in Group II, and SL317, known to be in Group III, in the phylogeny tree. The tomato outbreak strain C4.2 used in this study was placed in sv. Newport Group III, consistent with a previous phylogenetic analysis (Cao et al., 2013). Based on its position in the phylogenetic tree, it appears to be a typical representative of the sv. Newport Group III.

### Comparison of the *Salmonella* sv. Typhimurium and sv. Newport Pan-Genomes

The association of multiple strains of sv. Newport with recurrent produce-related outbreaks and the lack of such a strong association for sv. Typhimurium, coupled with the availability of parallel tools for the functional genomics characterization of the interactions of these organisms with diverse hosts, offers an opportunity to address the hypothesis that sv. Newport strains might have additional genes associated with success in tomatoes. Within 1526 *Salmonella* genomes that passed phylogeny quality controls, gene prediction using Prokka identified 31,675 gene orthologs. These data were used to build a pan-genome matrix (**Supplementary Table S1**) containing gene orthologs present in each genome. While sv. Typhimurium exhibited a small standard deviation in the number of genes per genome, the sv. Newport strains contained genomes that had up to 2,000 additional genes, primarily due to prophage and plasmids.

*Salmonella* sv. Typhimurium had an average of 4,628 genes per genome, while sv. Newport Group II had 4,554 genes per genome and sv. Newport Group III had 4,413 genes per genome (**Supplementary Figure S1**). The presence and absence gene profiles of each group were visualized by Principal Coordinate Analysis (**Figure 1B**). Principal Coordinate Analysis clustered the sv. Newport Groups II/III and sv. Typhimurium in two distinct groups separated by the first principal coordinate, showing that the difference among serovars corresponded to the presence and absence of genes, not only to serological or SNPs differences. The strain Newport C4.2, used in our model for tomato colonization, was within the cluster of sv. Newport Group III, confirming that its genetic profile is similar to most sv. Newport isolates.

**FIGURE 2:**
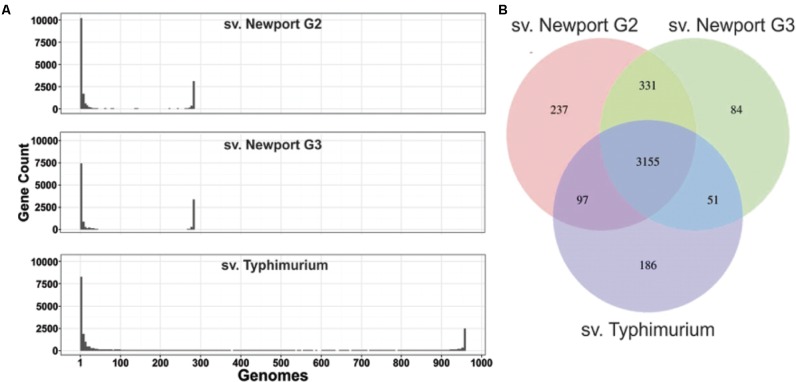
*Salmonella* core, shell and accessory genomes. **(A)** Number of genes per number of *Salmonella* sv. Newport Group II, sv. Newport Group III and sv. Typhimurium genomes. Many genes were found in only one or a few genomes (peaks on the left of each plot). The core genes, present in almost all genomes of each group, are represented by the peak on the right of each plot. **(B)** Venn diagram representing the shared and unique elements of *Salmonella* core genes (shared by >95% of all members of each group) among the groups studied.

Core and accessory genome analyses revealed similar genome structures for sv. Newport and Typhimurium. To further characterize the differences between *Salmonella* genomes, we assessed their core and accessory genome content. We used the definition of “core genome” as the genes shared by more than 95% of all the genomes per group, and “accessory genome” as the genes shared by less than 5% of the genomes per group. All the genes between these two classifications are defined as “shell genome.” In sv. Newport Group II and sv. Newport Group III, 61–68% of all identified gene orthologs were part of the accessory genome and 11–12% of all identified gene orthologs were in the core genome (**Figure 2A**). The relative abundance of genes in the accessory genome in relation to the core genome was similar in the three *Salmonella* groups tested in this study. Moreover, these data showed that most of the *Salmonella* genes identified in the groups investigated here were in the accessory genome. The number of genes in the core genome was similar within all groups, ranging from 3,489 to 3,820 genes. All groups shared 3,155 genes (90%) of their core genomes. The sv. Newport groups shared consistently more of their core genomes than they shared with sv. Typhimurium (**Figure 2B**).

We also investigated whether the serovars’ pan-genomes are open or closed according to the definition of (Medini et al., 2005; Tettelin et al., 2005). Using power-law regression, we found that all pan-genomes of *Salmonella* serovars analyzed in this study are closed, with an α parameter of 2.92, 2.94, and 2.39 (**Supplementary Figure S2**) for sv. Newport Group II, sv. Newport Group III and sv. Typhimurium, respectively. All regression curves exhibited similar slopes, although they reached saturation of new genes per genome at different points (around 20 for sv. Newport Group II and Group III, and 10 for sv. Typhimurium, **Supplementary Figure S2**). The genus *Salmonella* was previously identified as a closed genome species (Jacobsen et al., 2011).

The characterization of the presence/absence profile of genes in these genomes allowed the identification of 724 group-specific genes in the sv. Newport Group III shell genome and 84 group-specific genes unique to its core genome, compared with sv. Typhimurium. Further analysis was performed to determine if any of these genes were part of the genetic basis of the adaptation of the Group III Newport C4.2 strain to plant colonization.

### Transposon Insertion Sequencing Identifies Functions Required for Persistence in Tomatoes

To identify genome regions that encode functions important in colonization of tomato pericarps, we employed a library of transposon insertion mutants in sv. Newport C4.2, using the same screening methods applied before for sv. Typhimurium ATCC 14028 (de Moraes et al., 2017). The reads obtained from the transposon library screening were used to reconstruct the sv. Newport C4.2 genome (NCBI) using the RAST package. The fully annotated genome was deposited (see foot note text 1, username:guest, password:guest), and a comprehensive CSV file with locus tags, transposon insertion counts and orientation, and read counts is presented in **Supplementary Table S2**.

**FIGURE 3:**
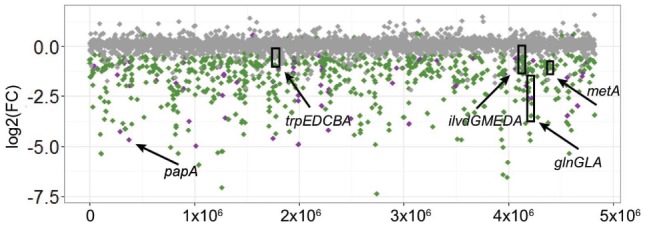
Change in abundance of *Salmonella* C4.2 loci after screening in tomatoes. Relative abundance of transposon insertions in loci after the incubation was compared to the initial inoculum. The position of each locus on the *y*-axis represents log_2_(Fold Change) of relative abundance and on the *x*-axis represents a physical position on the *Salmonella* chromosome. Loci with a significant change in abundance (FDR < 0.1) are shown in green when shared between *S.* Newport C4.2 and *S.* Typhimurium ATCC 14028 and purple when unique to *S.* Newport C4.2. Loci with an FDR > 0.1 are shown in gray. Arrows point to loci and operons targeted for further experiments.

We mapped 4,811 coding sequences disrupted by the transposon, and mutants in 796 of these had a significant change in their abundance after growth in tomato wounds (FDR < 0.1). 781 of these mutants displayed reduced fitness and 15 had increased fitness (**Figure 3**). Most of the genes (689) whose disruption led to a reduction of fitness were shared by sv. Newport C4.2 and sv. Typhimurium ATCC 14028.

**FIGURE 4:**
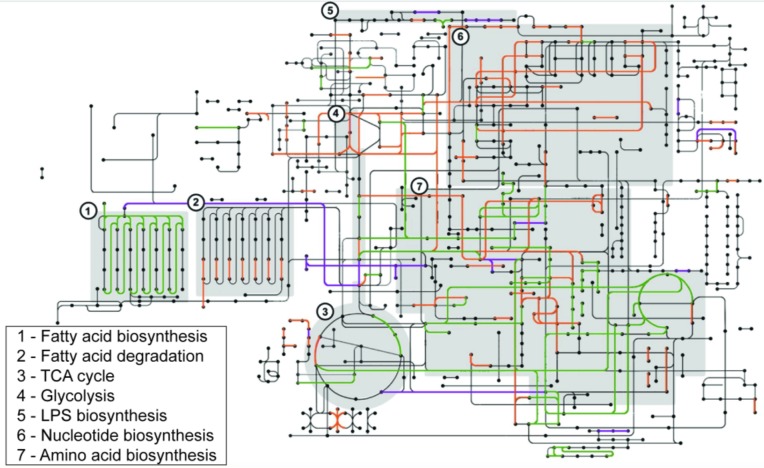
Comparison of functions required for colonization of tomatoes between *S.* Newport C4.2 and *S.* Typhimurium ATCC 14028. *S*. Newport C4.2 metabolic pathways involved in tomato colonization are shown in purple, *S.* Typhimurium ATCC 14028 metabolic pathways involved in tomato colonization are shown in orange, and required pathways shared by both strains are shown in green. Pathways were identified as required for tomato colonization if the corresponding mutants were less represented in the output pool [log_2_(FC) < -0.5, FDR < 0.1]. Key metabolic steps are shown as shaded boxes. KEGG Orthology terms for the protein sequences corresponding to the underrepresented mutants were retrieved using BlastKOALA and mapped using KEGG Mapper. False color overlays were imposed in Adobe Photoshop 2014.

To understand the cellular functions employed by sv. Newport C4.2 to colonize tomatoes, we identified the metabolic functions involved in this interaction. We mapped the genes identified as required for full fitness in pericarps and retrieved their COGs using the BlastKOALA web interface. Results were plotted against the sv. Newport C4.2 metabolic map. These results were compared with the outcomes of the sv. Typhimurium ATCC 14028 transposon insertion screening in a meta-analysis, with the goal of identifying potential serovar-specific factors. The comparison was done by overlapping the sv. Newport C4.2 and sv. Typhimurium ATCC 14028 metabolic maps. The main functions required for sv. Newport C4.2 colonization of tomato pericarps involved biosynthetic pathways such as amino acid and LPS biosynthesis, fatty acid catabolism and glycolysis. These same pathways were also required by sv. Typhimurium ATCC 14028 (**Figure 4**). The shared metabolic requirements by sv. Newport C4.2 and sv. Typhimurium ATCC 14028 corroborate the earlier conclusion that *Salmonella* relies on its robust and diverse metabolism to fully colonize tomatoes (de Moraes et al., 2017).

**FIGURE 5:**
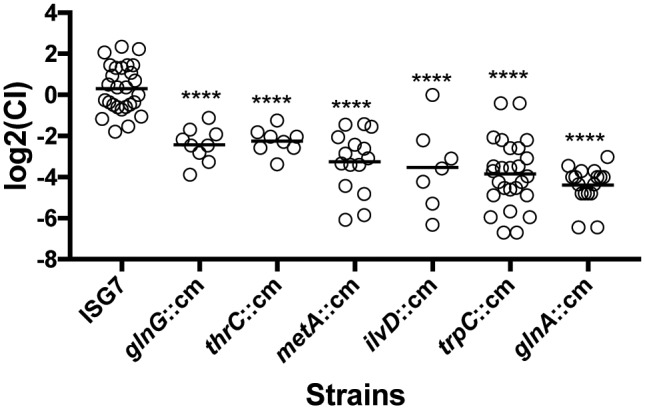
Competitive fitness of isogenic mutants involved in amino acid biosynthesis. Approximately 10^3^ CFUs of a mix of the wild type and an isogenic mutant (1:1) were inoculated into each tomato, followed by 7-day incubation at 22°C. The Competitive Index CI was calculated using the formula (MUTout:WTout)/(MUTin:WTin). The replicates were obtained from six tomato fruits with three inoculation sites per fruit. Each dot represents a single replicate and the black bar represents the mean log_2_(CI) of all replicates. Statistical significance was established by comparing CI’s using ANOVA and Tukey’s *post hoc* test. Asterisks represent strains that are statistically different from the neutral mutant ISG7 (*P*-value < 0.05).

Biosynthesis of amino acids by *Salmonella* during its interaction with tomatoes is of special interest for food safety. Different tomato cultivars are known to differ in the amounts of amino acids that accumulate within fruit during ripening (DiLeo et al., 2011; Osorio et al., 2011), and tomato genotypes with different amino acid profiles were shown to support different levels of *Salmonella* growth (Marvasi et al., 2014). To confirm the results obtained from the transposon insertion screening, we constructed isogenic mutants in the genes associated with amino acid biosynthesis and used these mutants to evaluate their fitness during tomato colonization in competition experiments against their parental strain. The genes selected were *thrC*, *metA*, *ilvD*, *trpC*, and *glnA*, involved in the biosynthesis of threonine, methionine, branched amino acids, tryptophan, and glutamine (respectively), and *glnG*, which codes for the master nitrogen regulator. Competition assays confirmed the results obtained from the transposon library screening. All isogenic mutants tested had a severe defect in fitness as estimated by their competitive indices (CI). The *glnA* isogenic mutant had the most severe defect in fitness [log_2_(CI) = -4.4], while the strain lacking the global nitrogen regulator had the smallest reduction of fitness [log_2_(CI) = -2.4] (**Figure 5**). The competition assays established that amino acid biosynthesis is a fundamental feature that confers an advantage for *Salmonella* to colonize tomato pericarps.

The main point of divergence between the sv. Newport C4.2 and sv. Typhimurium ATCC 14028 metabolic requirements for tomato colonization was the biosynthesis of nucleotides (**Figure 4**). Both purine and pyrimidine synthesis pathways identified as needed for efficient colonization by sv. Typhimurium ATCC 14028 at high titer were not under selection in the sv. Newport C4.2 transposon insertion screening at similar titers, indicating that *S.* Newport obtains sufficient purines and pyrimidines by scavenging in this environment. Notably, purine and pyrimidine biosynthesis was required by sv. Typhimurium ATCC 14028 even when inoculation was performed with a 10,000-fold lower titer (de Moraes et al., 2017). This result suggests that sv. Newport C4.2 could have a more efficient scavenging system for purines and pyrimidines, which could be advantageous for this strain during plant colonization.

### Phenotypic Analysis of the Genes Unique to sv. Newport Reveals Potential Adaptions to Persistence in Plants

**FIGURE 6:**
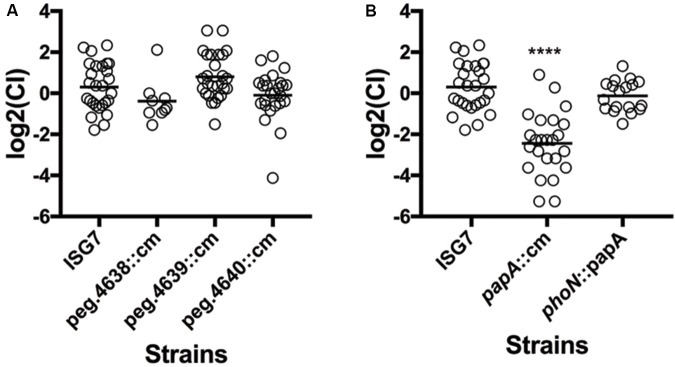
Competitive fitness of isogenic mutants of genes unique to *S.* Newport C4.2. The Competitive Index CI was calculated using the formula (MUTout:WTout)/(MUTin:WTin) and inoculation and statistical analysis was done as described before. **(A)** CI of isogenic mutants for peg.4638, peg.4639, and peg.4640. **(B)** CI of isogenic mutants for *papA* and the complemented isogenic mutant *ΔpapA phoN::papA*.

The main objective of this work was to explore the hypothesis that the sv. Newport C4.2 isolate from a tomato outbreak has additional genetic features not present in Typhimurium that enable it to colonize plants. Many sv. Newport-specific genes under selection had functions that could not be identified by BlastKOALA or Pfam. A few of those genes had putative metabolic functions. The locus peg.4637-peg.4641 is composed of genes that are absent from other *Salmonella* genomes and code for predicted proteins with unknown functions and a D-glucuronate permease (peg.4640). Since D-glucuronate is present in tomato fruit, we hypothesized that the reduction of fitness resulting from transposon insertions in this region indicates that *Salmonella* may be using D-glucuronate permease to scavenge D-glucuronate to proliferate in tomato pericarps. We therefore explored the role of this region during tomato colonization using a competition assay with isogenic mutants for the genes peg.4638, peg.4639, and peg.4640. The competitive indices for these isogenic mutants were not reduced when compared to ISG7, a strain that carries a neutral mutation (**Figure 6A**). However, this competition assay was performed with an inoculation titer thousands of fold lower than when the phenotype was observed in the transposon screen. It remains possible that this nutrient is used by *Salmonella* when the bacterial population is high.

**FIGURE 7:**
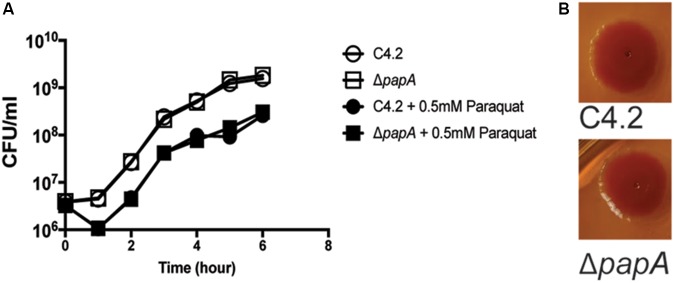
Comparison of growth and colony morphology between *S*. Newport C4.2 and its *papA* mutant. **(A)** Growth of strains in LB medium at 250 rpm and 37°C in the presence and absence of 0.5 mM paraquat. Three independent replicates were used per condition. **(B)** Colony morphology of *S*. Newport C4.2 and its *papA* mutant on Congo Red indicator plates. The smooth surface and edges show the absence of the rough and dry phenotype.

Another gene (peg.4132), coding for a small putative protein (44 amino acids), was present in the shell genome of *S. enterica* sv. Newport C4.2, and a corresponding mutant had a strong reduction of fitness [log_2_(FC) = -4.66] (**Figure 6B**). This gene was probably horizontally acquired: it is associated with a region containing mobile elements that includes remnants of phage genes, and it is located near a tRNA gene. The gene has a GC content of 40%, in contrast to the genome average of 52%. We further investigated the role of this gene during tomato colonization using competition assays with an isogenic mutant. The peg4132 gene was required for fitness of the strain *S. enterica* sv. Newport C4.2 in tomatoes [(log_2_(CI) = -2.44] (**Figure 6**). The mutant did not have a growth defect, displayed normal resistance to oxidative stress and was indistinguishable from the wild type when tested for the *rdar* phenotype (**Figure 7**). Due to its potential requirement for the colonization of plants, we named peg4132 “*papA”* (**P**lant **A**ssociated **P**rotein **A**).

To exclude the possibility that the reduction of fitness in this mutant in tomatoes was a result of a polar mutation, *papA* was cloned with its native promoter region in the vector pKD3 and then reinserted in the *phoN* locus in the *ΔpapA* strain, creating a complemented *papA* strain with a single copy of the gene, stably maintained on the chromosome. The *phoN* locus was previously shown to be neutral during tomato colonization (Cox et al., 2013). The colonization fitness measured by the competitive index (CI) was not significantly different between the wild type and complemented *papA* strains (**Figure 6B**), corroborating that *papA* is a factor required for full fitness in sv. Newport C4.2 colonization of tomato pericarps.

We analyzed the distribution of *papA* in the *Salmonella* genomes, and determined that this gene was exclusive to Newport Group III, where it was found in 71 genomes out of 306, most of them closely associated in one clade. Using BlastP over the NCBI microbial database, we found potential orthologs of *papA*. Interestingly, many of these orthologs were in other members of the Enterobacteriaceae family that have a lifestyle associated with soil and plants, including *Pectobacterium* ssp. and *Dickeya* ssp, suggesting that this gene may be involved in persistence in or on plants.

## Author Contributions

MdM, MM, and MT conceived the study and designed the experiments. MdM, EBS, ISG, and PD conducted the computational analysis. MM conceived, designed and oversaw Tn-Seq experiments and the Tn-Seq data analyses. MdM and WC conducted experimental work. MdM, MT, SP, and MM wrote the paper.

## Conflict of Interest Statement

The authors declare that the research was conducted in the absence of any commercial or financial relationships that could be construed as a potential conflict of interest.

## Footnotes

1 http://rast.nmpdr.org/rast.cgi?page=JobDetails&job=211518

## Supplementary Material

FIGURE S1 Number of genes per genome. The total number of genes per genome was plotted for the groups sv. Newport Gil, sv. Newport Group TIT and sv. Typhimurium. The upper whisker extends to the highest value within 1.5 times the interquartile range. The lower whisker extend to the lowest value within 1.5 times the interquartile range. Outliers were defined as values higher than 1.5× interquartile range ± third quartile, or lower than 1.5 ± first quartile, and were plotted as dots. The thick black line within the boxes represents the mean.

FIGURE S2 Total number of genes and new genes per genome obtained using random permutations. **(A,C,E)** Total number of genes per genome added. **(B,D,F)** New genes per genome and α and 𝜃 values from power law regression. **(A,B)** sv. Newport Group II. **(C,D)** sv. Newport Group III. **(E,F)** sv. Typhimurium.

TABLE S1 Pangenome matrix containing genomes ID, metadata, and presence/absence profile of genes.

TABLE S2 Genome Annotation of Newport C4.2 plus Tn5 fitness screening data compared to Typhimurium.

## References

[B1] Andrews S. Krueger F. Segonds-Pichon A. Biggins L. Virk B. Dalle-Pezze P. (2015). Babraham: Babraham Institute.

[B2] Bankevich A. Nurk S. Antipov D. Gurevich A. A. Dvorkin M. Kulikov A. S. (2012). SPAdes: a new genome assembly algorithm and its applications to single-cell sequencing. 19 455–477. 10.1089/cmb.2012.0021 PMC3342519 22506599

[B3] Barrow P. A. Lovell M. A. (1988). The association between a large molecular mass plasmid and virulence in a strain of *Salmonella*-Pullorum. 134 2307–2316. 10.1099/00221287-134-8-2307 3253408

[B4] Barrow P. A. Simpson J. M. Lovell M. A. Binns M. M. (1987). Contribution of *Salmonella* Gallinarum large plasmid toward virulence in fowl typhoid. 55 388–392. 3804442 10.1128/iai.55.2.388-392.1987 PMC260339

[B5] Bennett S. D. Littrell K. W. Hill T. A. Mahovic M. Behravesh C. B. (2015). Multistate foodborne disease outbreaks associated with raw tomatoes, United States, 1990-2010: a recurring public health problem. 143 1352–1359. 10.1017/S0950268814002167 25167220 PMC9507195

[B6] Cao G. Meng J. Strain E. Stones R. Pettengill J. Zhao S. (2013). Phylogenetics and differentiation of *Salmonella* Newport lineages by whole genome sequencing. 8:e55687. 10.1371/journal.pone.0055687 23409020 PMC3569456

[B7] Cox C. E. Mcclelland M. Teplitski M. (2013). Consequences of disrupting *Salmonella* AI-2 signaling on interactions within soft rots. 103 352–361. 10.1094/PHYTO-09-12-0237-FI 23324045

[B8] Datsenko K. A. Wanner B. L. (2000). One-step inactivation of chromosomal genes in *Escherichia coli* K-12 using PCR products. 97 6640–6645. 10.1073/pnas.120163297 10829079 PMC18686

[B9] de Moraes M. H. Desai P. Porwollik S. Canals R. Perez D. R. Chu W. (2017). *Salmonella* persistence in tomatoes requires a distinct set of metabolic functions identified by transposon insertion sequencing. 83 e3028-16. 10.1128/AEM.03028-16 28039131 PMC5311394

[B10] Desai P. T. Porwollik S. Long F. Cheng P. Wollam A. Bhonagiri-Palsikar V. (2013). Evolutionary genomics of *Salmonella enterica* subspecies. 4:e198-13. 10.1128/mBio.00198-13 23462113 PMC3604774

[B11] DiLeo M. V. Strahan G. D. Den Bakker M. Hoekenga O. A. (2011). Weighted correlation network analysis (WGCNA) applied to the tomato fruit metabolome. 6:e26683. 10.1371/journal.pone.0026683 22039529 PMC3198806

[B12] Hernandez-Reyes C. Schikora A. (2013). *Salmonella*, a cross-kingdom pathogen infecting humans and plants. 343 1–7. 10.1111/1574-6968.12127 23488473

[B13] Jackson B. R. Griffin P. M. Cole D. Walsh K. A. Chai S. J. (2013). Outbreak-associated *Salmonella enterica* serotypes and food commodities, United States, 1998-2008. 19 1239–1244. 10.3201/eid1908.121511 23876503 PMC3739514

[B14] Jacobsen A. Hendriksen R. S. Aaresturp F. M. Ussery D. W. Friis C. (2011). The *Salmonella enterica* pan-genome. 62 487–504. 10.1007/s00248-011-9880-1 21643699 PMC3175032

[B15] Letunic I. Bork P. (2016). Interactive tree of life (iTOL) v3: an online tool for the display and annotation of phylogenetic and other trees. 44 W242–W245. 10.1093/nar/gkw290 27095192 PMC4987883

[B16] Libby S. J. Adams L. G. Ficht T. A. Allen C. Whitford H. A. Buchmeier N. A. (1997). The spv genes on the *Salmonella* Dublin virulence plasmid are required for severe enteritis and systemic infection in the natural host. 65 1786–1792. 9125562 10.1128/iai.65.5.1786-1792.1997 PMC175217

[B17] Lindow S. E. Brandl M. T. (2003). Microbiology of the phyllosphere. 69 1875–1883. 10.1128/AEM.69.4.1875-1883.2003 PMC154815 12676659

[B18] Lindow S. E. Leveau J. H. (2002). Phyllosphere microbiology. 13 238–243. 10.1016/S0958-1669(02)00313-0 12180099

[B19] Marvasi M. Cox C. E. Xu Y. Noel J. T. Giovannoni J. J. Teplitski M. (2013a). Differential regulation of *Salmonella* Typhimurium genes involved in O-antigen capsule production and their role in persistence within tomato fruit. 26 793–800. 10.1094/MPMI-09-12-0208-R 23489058

[B20] Marvasi M. Hochmuth G. J. Giurcanu M. C. George A. S. Noel J. T. Bartz J. (2013b). Factors that affect proliferation of *Salmonella* in tomatoes post-harvest: the roles of seasonal effects, irrigation regime, crop and pathogen genotype. 8:e80871. 10.1371/journal.pone.0080871 24324640 PMC3851777

[B21] Marvasi M. Noel J. T. George A. S. Farias M. A. Jenkins K. T. Hochmuth G. (2014). Ethylene signalling affects susceptibility of tomatoes to *Salmonella*. 7 545–555. 10.1111/1751-7915.12130 24888884 PMC4265073

[B22] Medini D. Donati C. Tettelin H. Masignani V. Rappuoli R. (2005). The microbial pan-genome. 15 589–594. 10.1016/j.gde.2005.09.006 16185861

[B23] Noel J. T. Arrach N. Alagely A. Mcclelland M. Teplitski M. (2010). Specific responses of *Salmonella enterica* to tomato varieties and fruit ripeness identified by *in vivo* expression technology. 5:e12406. 10.1371/journal.pone.0012406 20824208 PMC2930847

[B24] Nugent S. L. Meng F. Martin G. B. Altier C. (2015). Acquisition of iron is required for growth of *Salmonella* spp. in tomato fruit. 81 3663–3670. 10.1128/AEM.04257-14 25795672 PMC4421055

[B25] Osorio S. Alba R. Damasceno C. M. Lopez-Casado G. Lohse M. Zanor M. I. (2011). Systems biology of tomato fruit development: combined transcript, protein, and metabolite analysis of tomato transcription factor (nor, rin) and ethylene receptor (Nr) mutants reveals novel regulatory interactions. 157 405–425. 10.1104/pp.111.175463 21795583 PMC3165888

[B26] Page A. J. Cummins C. A. Hunt M. Wong V. K. Reuter S. Holden M. T. (2015). Roary: rapid large-scale prokaryote pan genome analysis. 31 3691–3693. 10.1093/bioinformatics/btv421 26198102 PMC4817141

[B27] Parks D. H. Imelfort M. Skennerton C. T. Hugenholtz P. Tyson G. W. (2015). CheckM: assessing the quality of microbial genomes recovered from isolates, single cells, and metagenomes. 25 1043–1055. 10.1101/gr.186072.114 25977477 PMC4484387

[B28] Sangal V. Harbottle H. Mazzoni C. J. Helmuth R. Guerra B. Didelot X. (2010). Evolution and population structure of *Salmonella enterica* serovar Newport. 192 6465–6476. 10.1128/JB.00969-10 20935094 PMC3008538

[B29] Seemann T. (2014). Prokka: rapid prokaryotic genome annotation. 30 2068–2069. 10.1093/bioinformatics/btu153 24642063

[B30] Teplitski M. Barak J. D. Schneider K. R. (2009). Human enteric pathogens in produce: un-answered ecological questions with direct implications for food safety. 20 166–171. 10.1016/j.copbio.2009.03.002 19349159

[B31] Teplitski M. de Moraes M. (2018). Of mice and men....and plants: comparative genomics of the dual lifestyles of enteric pathogens. 10.1016/j.tim.2018.02.008 [Epub ahead of print]. 29502873

[B32] Tettelin H. Masignani V. Cieslewicz M. J. Donati C. Medini D. Ward N. L. (2005). Genome analysis of multiple pathogenic isolates of *Streptococcus agalactiae*: implications for the microbial “pan-genome”. 102 13950–13955. 10.1073/pnas.0506758102 16172379 PMC1216834

[B33] Wiedemann A. Virlogeux-Payant I. Chausse A. M. Schikora A. Velge P. (2014). Interactions of *Salmonella* with animals and plants. 5:791 10.3389/fmicb.2014.00791 PMC4301013 25653644

[B34] Zaragoza W. J. Noel J. T. Teplitski M. (2012). Spontaneous non-rdar mutations increase fitness of *Salmonella* in plants. 4 453–458. 10.1111/j.1758-2229.2012.00364.x 23760832

[B35] Zheng J. Allard S. Reynolds S. Millner P. Arce G. Blodgett R. J. (2013). Colonization and internalization of *Salmonella enterica* in tomato plants. 79 2494–2502. 10.1128/AEM.03704-12 23377940 PMC3623171

