# Supplementary figures and images for: Genome-Wide Comparative Functional Analyses Reveal Adaptations of Salmonella sv. Newport to a Plant Colonization Lifestyle
Source: Front Microbiol. 2018 May 18;9:877. doi: 10.3389/fmicb.2018.00877 (PMC5968271; doi:10.3389/fmicb.2018.00877)

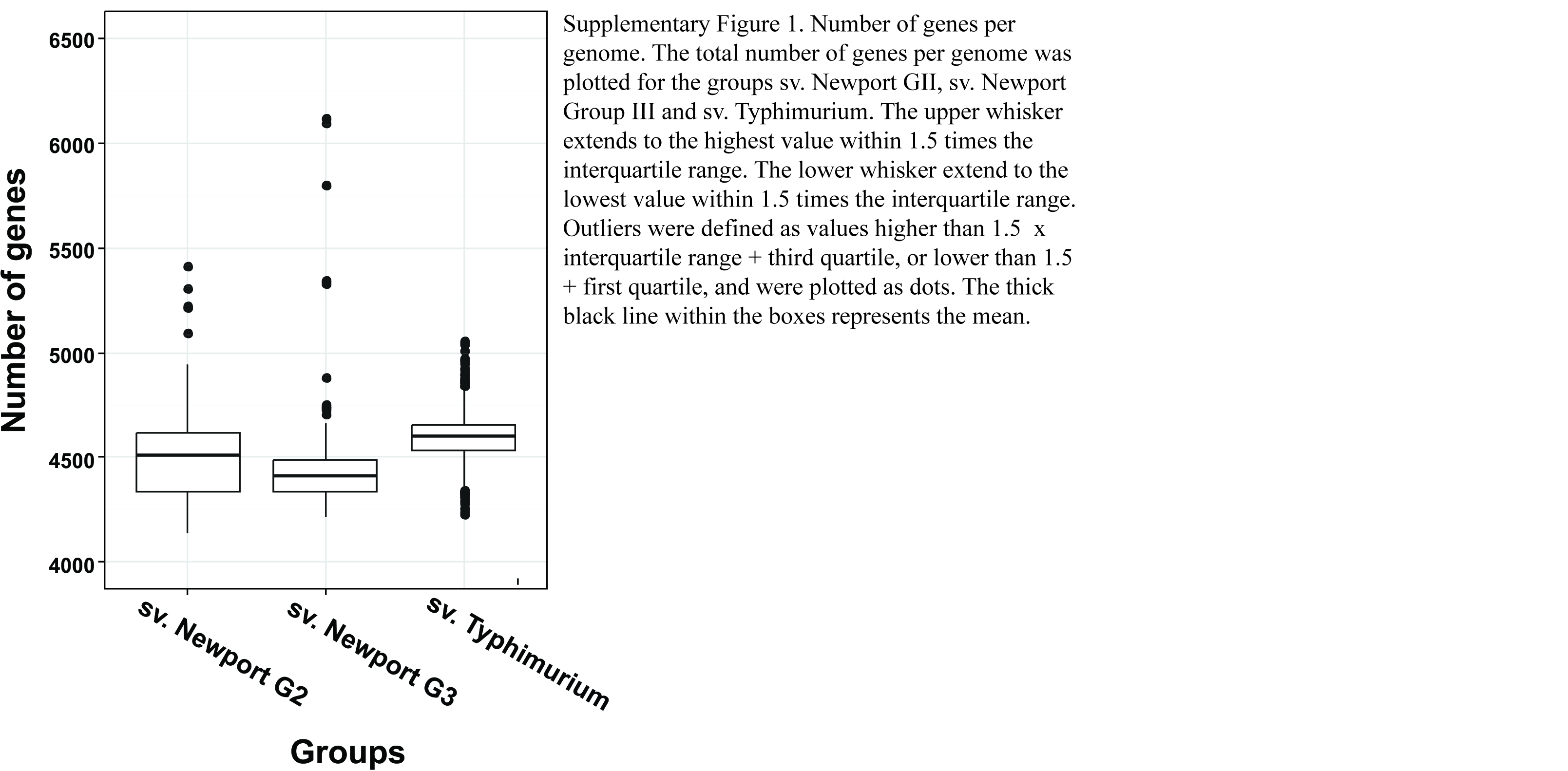

Supplement: FIGURE S1 — Number of genes per genome. The total number of genes per genome was plotted for the groups sv. Newport Gil, sv. Newport Group TIT and sv. Typhimurium. The upper whisker extends to the highest value within 1.5 times the interquartile range. The lower whisker extend to the lowest value within 1.5 times the interquartile range. Outliers were defined as values higher than 1.5× interquartile range ± third quartile, or lower than 1.5 ± first quartile, and were plotted as dots. The thick black line within the boxes represents the mean. [file Image_1.tif]

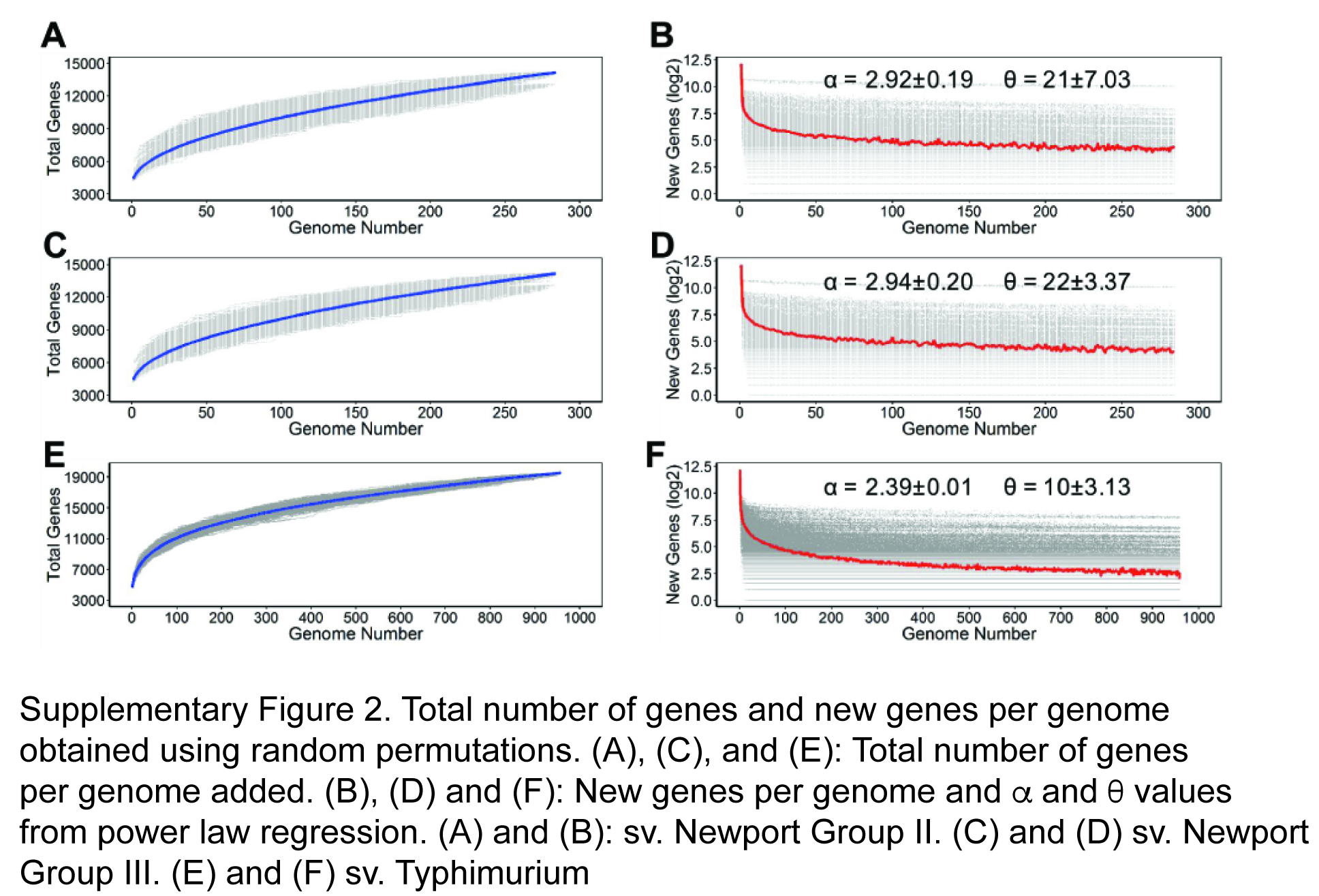

Supplement: FIGURE S2 — Total number of genes and new genes per genome obtained using random permutations. (A,C,E) Total number of genes per genome added. (B,D,F) New genes per genome and α and 𝜃 values from power law regression. (A,B) sv. Newport Group II. (C,D) sv. Newport Group III. (E,F) sv. Typhimurium. [file Image_2.tif]
